# Investigation of Confounding Factors in Measuring Tissue Saturation with NIRS Spatially Resolved Spectroscopy

**DOI:** 10.1007/978-3-319-91287-5_49

**Published:** 2018-04-16

**Authors:** Z. Kovacsova, G. Bale, S. Mitra, I. de Roever, J. Meek, N. Robertson, I. Tachtsidis

**Affiliations:** 9 Medical Physics & Biomedical Engineering, UCL, London, UK; 10 Department of Neonatology, Institute for Women’s Health, UCL, London, UK

## Abstract

Performing absolute measurements of tissue saturation of the brain with near-infrared spectroscopy (NIRS) is a clinically desirable brain monitoring tool. Tissue oxygenation index (TOI) is an indicator of absolute tissue mixed arterial and venous oxygen saturation, and can be calculated using a NIRS technique called spatially resolved spectroscopy (SRS). SRS instruments measure the change of light attenuation with distance by using multiple light source-detector distances at two or more wavelengths. The aim of the study is to use broadband NIRS SRS data to investigate the effects on the calculation of TOI of different parameters: wavelength selection, scattering dependence, source-detector distance, and resolving for water. In total, 55 neonates with hypoxic-ischemic encephalopathy were monitored using a broadband multi-distance continuous wave NIRS system; 172 datasets were recorded. Using a “Standard” approach, TOI values between 0 and 100% (“good”) were calculated in 157/172 datasets with a mean TOI of 50%. By changing the wavelength selection, the number of “good” data sets increases to 165/172 with a mean of 60%. Alteration of the dependence of scattering on wavelength acts as a constant which shifts the absolute value of TOI significantly (p < 0.05), demonstrating the importance of having a subject-appropriate estimation of scattering dependence. In general, changing the combination of source-detector distances does not significantly alter the TOI (the mean TOI ranges from 41% to 53%) which suggests that the algorithm is robust to different source-detector combinations. The study shows the broadband NIRS SRS algorithm gives the opportunity to explore the calculation of TOI and could further improve the measurement of tissue saturation in a clinical setting.

## Introduction

Near-infrared spectroscopy (NIRS)Broadband NIRS SRS Tissue saturation Spatially resolved spectroscopy (SRS) is a Broadband NIRS SRS which provides real-time information on the oxygenation and haemodynamics of tissue. It is based on the absorption of near-infrared light by haemoglobin; as the absorption properties of haemoglobin change with its oxygenation status, measuring the transmitted/reflected light from tissue informs on its oxygenation state of tissue. Using NIRS to monitor Broadband NIRS SRS in the brain is clinically desirable as it can inform on the balance between cerebral blood flow and oxygen supply. NIRS can measure brain tissue oxygen saturation StO_2_, also known as Tissue oxygenation index (TOI), which is the ratio of the concentration of oxyhaemoglobin HbO_2_ and total haemoglobin HbT = HbO_2_ + HHb, where HHb is deoxyhaemoglobin. One of the methods to measure absolute tissue oxygenation is spatially resolved spectroscopy (SRS). SRS Spatially resolved spectroscopy (SRS) are able to measure the change in light attenuation *A* with distance *ρ* by using two or more source-detector separations. A scaled absorption coefficient *kμ*_*a*_ is calculated using the slope of *A*:

$$ \mathrm{k}{\upmu}_{\mathrm{a}}=\frac{1}{3\left(1- h\lambda \right)}{\left(\ln (10)\frac{\partial A\left(\uplambda \right)}{\partial \rho }-\frac{2}{\uprho}\right)}^2 $$

where *h* is the gradient of the transport scattering coefficient *μ*_*s*_ *’* with wavelength *λ*; *μ*_*s*_ *'=k(1-hλ)*. Scaled concentration of haemoglobin *k*[HbO_2_] and *k*[HHb] are quantified; the Broadband NIRS SRS *k* (which is unknown from the measurement) cancels itself when expressing oxygenation in terms of TOITissue oxygenation index (TOI), i.e. TOI= *k*[HbO_2_]/*k*[HbT] [1].

## Method

The data in this study were previously published by Bale et al. [2]. A broadband multi-distance continuous wave NIRS system, Broadband NIRS SRS CYtochrome Research Instrument and appLication (CYRIL) (source-detector distances: 30, 25, 20, 15 mm, 770–906 nm) [3], was used to monitor the right frontal hemisphere of neonates with hypoxic-ischaemic encephalopathy during the first 4 days of life. The patients were diagnosed with severe or moderate brain injury and all were treated with therapeutic hypothermia for 48 h. Data were recorded from 55 neonates and yielded 172 separate datasets (durations from 10 min to 18 h). To minimize computational burden, datasets were cut to be 600 s long; the time window was 200–799 s. The first 199 s of the measurement were excluded to avoid set-up errors. The “Standard” SRS methodSpatially resolved spectroscopy (SRS) Standard SRS method (described below) produced, in some cases, TOITissue oxygenation index (TOI) values which were negative or above 100%. We now attempt to correct these values by investigating the SRS algorithm and how the input parameters influence TOITissue oxygenation index (TOI). The different approaches to resolve TOI were:

- Approach 0, “StandardSpatially resolved spectroscopy (SRS) Standard SRS method”: This SRS approach resolved for only haemoglobin chromophores from *kμ*_*a*_; the wavelength dependence of the scattering coefficient was *h* = 0.00063 mm^−1^ nm^−1^ [1, 4]; the entire broadband spectrum of the NIRS data (770–906 nm) was used; and all four detectors were used to quantify the attenuation slope.
- Approach 1, “Water”: This approach additionally resolved for the water concentration from *kμ*_*a*_, i.e. water was treated as a third chromophore like HbO_2_ and HHb. It does not obtain an absolute measure of the concentrations as *k* is unknown.
- Approach 2, “Scattering”: The dependence of scattering on wavelength as described by *h* was modified. The “Standard” approach uses *h* = 0.00063 mm^−1^ nm^−1^ measured by Matcher et al. on adults [2] which is used in commercial systems [4]. In this approach, two other values are used. Highton et al. measured a value of *h* = 0.00085 mm^−1^ nm^−1^ in adults with traumatic brain injury [5]. Kurata et al. measured optical properties of the neonatal brain [6], a value of *h* = 0.00048 mm^−1^ nm^−1^ was calculated from the data for term infants using the method described by Matcher et al. [4].
- Approach 3, “Distance”: The number and combination of source-detector separations was adjusted. A minimum of two source-detector distances are necessary for SRS; increasing their number leads to a more accurate approximation of the gradient of attenuation with distance. In this approach, the effect of using less detectors and applying various combinations of the detectors was studied; there were ten possible combinations.
- Approach 4, “Wavelengths”: The “Standard” approach uses the full broadband spectrum; however, it is possible to measure TOITissue oxygenation index (TOI) with a minimum of two wavelengths. The selected wavelengths were 775, 810, 850, 905 nm or 775, 810, 850 nm which were chosen because they are used in commercial systems; NIROSpatially resolved spectroscopy (SRS) Standard SRS method 300, NIRO 200 (Hamamatsu Photonics, Japan).

Each approach was investigated independently from the others; the other parameters remained as in the “Standard” SRS approachSpatially resolved spectroscopy (SRS) Standard SRS method. Approaches “Scattering”, “Distance” and “Wavelength” have several sub-approaches. In total, there were 16 approaches resulting in 16 TOITissue oxygenation index (TOI) values calculated from each dataset.

The resulting TOIsTissue oxygenation index (TOI) were averaged over the time window and compared to the “Standard” average TOI. A TOI value was considered to be “good” if it was between 0% and 100%.^1^ The number of “good” TOIs was counted for all approaches and sub-approaches. Minimum and maximum TOIsTissue oxygenation index (TOI) were found for all “good” TOIsTissue oxygenation index (TOI) out of the 172 datasets. Datasets in which all 16 TOI values were “good” were further analysed using Broadband NIRS SRS (IBM 2016). A Sign test was applied to evaluate differences between “Standard” and every other approach, significance threshold p < 0.05. Spearman’s rho (*ρ*_*s*_) was calculated between the “Standard” TOIsTissue oxygenation index (TOI) and every other approach to assess their correlation. The combination of analysing the statistical difference between approaches and their correlation can give us information about their similarity, for example:

- if *ρ*_*s*_ is low and there is a significant difference between the approaches, then these are not similar.
- when *ρ*_*s*_ is high and there is a significant difference between the approaches, then these are highly associated but have different absolute TOITissue oxygenation index (TOI) values.
- if *ρ*_*s*_ is high and there is not a significant difference between the approaches then these approaches are similar.

## Results

Table 1 shows the calculated TOITissue oxygenation index (TOI) values with the percentage of “good” TOI values for each SRS approach. The number of datasets which give “good” TOIs changes per approach; the most successful one is “Wavelengths (775,810,850 nm)” with 165/172 “good” TOIs and the least successful is “Distance (15,20 mm)” with 95/172.

**Table 1:** Number of “good” TOITissue oxygenation index (TOI) values from the 172 datasets calculated using the different approaches

Approach	Sub-approach	Good data sets (/172)	Success [%]	Mean TOI [%]	Min TOI [%]	Max TOI [%]	SD [%]
0: Standard		157	91	50	6	96	16
1: Water		161	94	62	28	96	10
2: Scattering (mm^−1^ nm^−1^)	0.00085	143	83	66	22	96	15
	0.00048	159	92	46	7	92	17
3: Distance (mm)	20,15	95	55	41	6	96	24
	25,15	152	88	53	16	99	18
	30,15	154	90	51	12	91	17
	25,20	158	92	50	6	95	11
	30,20	162	94	49	3	92	14
	30,25	102	59	45	4	86	17
	25,20,15	151	88	53	16	96	18
	30,20,15	154	90	50	8	89	17
	30,25,15	149	87	51	6	97	18
	30,25,20	161	94	49	3	92	14
4: Wavelength (nm)	775,810,850,905	163	95	53	14	90	14
	775,810,850	165	96	60	28	95	11

Mean TOI, Min TOI, Max TOI and the standard deviation (SD) are calculated only from the “good” values

**Fig. 1:**
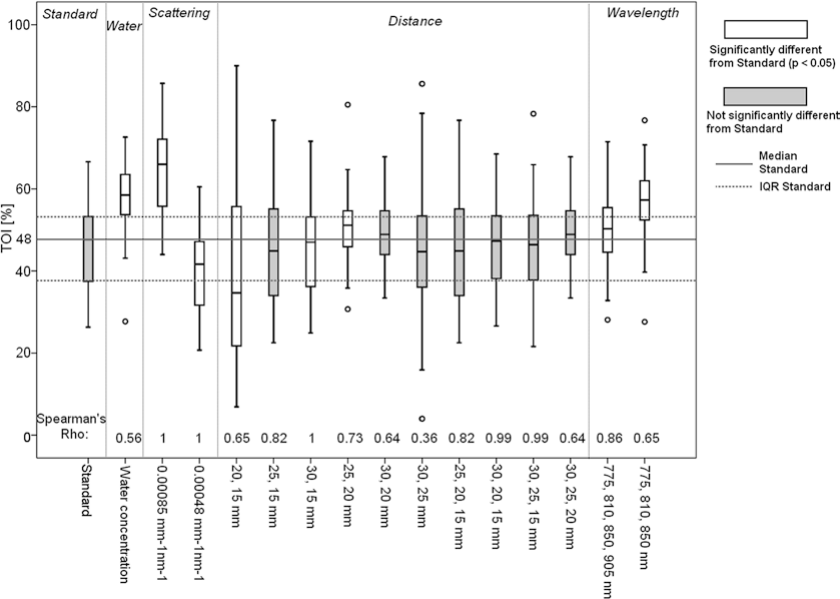
Boxplot of median TOIsTissue oxygenation index (TOI) for the 48 datasets in which all approaches give “good” TOI values. The full line is the median of the “Standard” approach and the dotted lines are its inter-quartile range. Circles are outliers. White boxes indicate significant difference (p < 0.05) from the “Standard” approach. Spearman’s Rho indicates the correlation between the “Standard” approach and other approaches

For 48 out of 172 datasets all 16 approaches gave “good” TOITissue oxygenation index (TOI) values. Figure 1 shows a boxplot of the median TOI values with Spearman’s Rho for each approach.

The Broadband NIRS SRS Sign test showed that eight approaches gave significantly different results from the “Standard” (p < 0.05). Those were: “Water”, “Scattering” (both *h* values), “Distance” (20,15 mm; 30,15 mm; 25,20 mm) and “Wavelengths” (both sets). Spearman’s Rho was below 0.7 in six different instances, which suggests low association with the “Standard” approach: “Water” (*ρ*_*s*_ = 0.56), “Distance” (*ρ*_*s*_ (20,15 mm) = 0.65, *ρ*_*s*_ (30,20 mm) = 0.64, *ρ*_*s*_ (30,25 mm) = 0.36, *ρ*_*s*_ (30,25,20 mm) = 0.64) and “Wavelength” (*ρ*_*s*_ (775,810,850 nm) = 0.65).

## Discussion

The aim of this study was to investigate the SRS algorithm using a multi-distance broadband NIRS device. The parameters investigated were: resolving for water, the scattering dependence on wavelength, source-detector distance combinations and wavelength selection. We have shown that TOITissue oxygenation index (TOI) values vary significantly depending on the SRS approach.

To investigate the effect of changing the SRS approach, we quantifiedBroadband NIRS SRS how many times a “good” TOITissue oxygenation index (TOI) value (between 0 and 100%) was estimated per dataset (Table 1). Changing the approach sometimes changes the TOI value from negative/above 100% to what we call “good” TOI (between 0% and 100%). Even within “good” TOIsTissue oxygenation index (TOI), the range of achievable values is broad. This is further explored by focusing on 48 datasets in which all 16 approaches result in “good” TOI – the median values significantly differ from “Standard” in the majority of approaches (Fig. 1). It is only the “Distance” approach, which gives similar results to “Standard” (p > 0.05 in 7/10 distance combinations), which suggests that the algorithm is robust to different source-detector combinations.

The combination of the Spearman’s Rho and the Broadband NIRS SRS demonstrated low similarity between the “Standard” approach and: “Water”, “Distance” (20, 15 mm) and “Wavelength” (775,810,850 nm) approaches. However the approaches “Distance” (30,25,15 and 30,20,15 mm) had the most similar results to the “Standard” approach.

The approach “Broadband NIRS SRS” shows that the selection of wavelength has a significant impact on the resulting TOITissue oxygenation index (TOI); this is important as different commercial brain oximeters (that apply the SRS method) use different wavelengths. For example OxyMon (Artinis Medical Systems, the Netherlands) uses 765 and 855 nm, but NIRO-200NX (Hamamatsu, Japan) uses 735, 810 and 850 nm.

Significant shifts in TOITissue oxygenation index (TOI) were caused by changing *h*; each value of *h* was acquired in a different patient group: 0.00063 mm^−1^ nm^−1^ in 7 healthy adults, 0.00085 mm^−1^ nm^−1^ in 21 brain injured adults and 0.00048 mm^−1^ nm^−1^ in 8 full-term neonates. This demonstrates the importance of having a subject-appropriate estimation of wavelength dependence of scattering. It has been shown that scattering properties change with age and pathology [6–8]; more investigations of the scattering dependence on wavelength and its behaviour during pathology are needed.

A broadband NIRS multi-distance device can provide additional information to improve the robustness of the SRS Broadband NIRS SRS of TOITissue oxygenation index (TOI), as it gives insight into the individual parameters in the algorithm and helps quantify their importance and impact. The limitation of this study is that the brain oxygenation of the studied patients is unknown and thus there is no physiological comparison.

The next steps include studying the effect on TOITissue oxygenation index (TOI) by combining several approaches at once; and doing this using a dynamic blood phantom that allow us to control oxygenation (similar to a phantom described in [9]). We will do this while simultaneously measuring chromophore concentrations and oxygenation with a Time-resolved system (TR). TR systems measure absolute concentrations of chromophores by resolving absorption and scattering [10]. This will enable us to further study and evaluate the effect of each approach and possibly identifying an optimal combination of parameters.

## Footnotes

1 This was used to exclude physiologically impossible values. At this stage of the study, it was not evaluated whether the TOI was a correct estimation of the brain oxygenation of the patient.
